# Polymorphisms in *SELE* Gene and Risk of Coal Workers' Pneumoconiosis in Chinese: A Case-Control Study

**DOI:** 10.1371/journal.pone.0073254

**Published:** 2013-09-16

**Authors:** Ting Wang, Xiaoming Ji, Chen Luo, Jingjing Fan, Zhiguo Hou, Minjuan Chen, Ruhui Han, Chunhui Ni

**Affiliations:** Department of Occupational Medicine and Environmental Health, School of Public Health, Nanjing Medical University, Nanjing, China; University of North Dakota, United States of America

## Abstract

**Background:**

Coal workers' pneumoconiosis (CWP) is characterized by chronic pulmonary inflammation and fibrotic nodular lesions that usually lead to progressive fibrosis. Inflammation is the first step in the development of CWP. E-selectin, an adhesion molecule, is involved in the development of various inflammatory diseases.

**Methods:**

We investigated the association between the functional polymorphisms in *SELE* and the risk of CWP in Han Chinese population. Three polymorphisms (T1880C/rs5355, T1559C/rs5368, A16089G/rs4786) in *SELE* were genotyped and analyzed in a case-control study with 697 CWP cases and 694 controls. The genotyping was based on the TaqMan method with the ABI 7900HT Real Time PCR system.

**Results:**

The *SELE* rs5368 CT genotype was associated with a significantly increased risk of CWP (OR = 1.28, 95% CI = 1.02–1.60, *P* = 0.03) relative to the CC genotype. The statistical analysis of classification and regression tree (CART) and multifactor dimensionality reduction (MDR) were used to predict the interactions among risk factors of CWP. The MDR analysis found that the best interaction model was the two-factor model that contains pack-years smoked and *SELE* rs5368 genotypes. For non-smokers, the CART analysis showed an increased risk of CWP for carriers of the *SELE* rs_5368 variant genotype compared with the common genotype (OR = 1.51; 95% CI = 1.11–2.05, *P* = 0.0069).

**Conclusion:**

The results suggest that the T1559C/rs5368 polymorphism and smoking are involved in the susceptibility to CWP. Further studies are warranted to validate these findings.

## Introduction

Coal workers' pneumoconiosis (CWP) is a chronic occupational lung disease caused by the long-term inhalation and deposition of coal mine dust. The dust triggers a persistent inflammatory response and generation of pro-inflammatory and pro-fibrotic mediators, eventually resulting in irreversible lung damage [1], [2]. In 2010, 87.42% of the total reported pneumoconiosis cases were attributed to occupation, and over 50% were related to underground mining [3]. Underground mining is associated with exposures to silica, metals, and coal dust generated during tunnel drilling, mining, roof bolting, and transportation. Most cases of CWP are caused by silica exposure.

Inhalation of respirable silica particles leads to lung injury and alveolar macrophage activation, which initiate inflammation. In the inflammation phase and subsequent fibrosis, various cells, including inflammatory cells, alveolar epithelial cells, mast cells, endothelial cells, and mesenchymal cells, form a complex network and interact with each other to promote the development of lung fibrosis by secreting cytokines, inflammatory mediators, and other bioactive substances [4]. Several epidemiological and experimental studies have been conducted to elucidate the pathogenesis of silicosis, but to date the cellular mechanisms that initiate and drive the processes of inflammation and fibrogenesis are not clear [4]–[7].

E-selectin, an 11-kD cell surface glycoprotein synthesized by endothelial cells, is an adhesion molecule of the selectin family. It mediates leukocyte-endothelial adhesion in various physiological and pathological settings [8], [9]. Its corresponding gene, *SELE* (CD62E; ELAM1), is located on human chromosome 1q22-q25 [10]. Recent studies have confirmed that E-selectin is involved in pulmonary diseases [11], [12]. Here we evaluated the frequency distributions of three *SELE* single nucleotide polymorphisms (SNPs) to explore the association between these polymorphisms and CWP. Two non-synonymous candidate SNPs, T1880C/rs5355 and T1559C/rs5368, are located in the exon region of the E-selectin gene and one SNP, A16089G/rs4786, is in the 3′UTR region of the E-selectin gene among all functioning SNPs in *SELE*, were chosen to be genotyped. These variants may provide clues to the pathogenesis of CWP [13]–[15].

## Materials and Methods

### Study subjects

In an ongoing study, 697 CWP patients and 694 controls were males and recruited from the coal mines of Xuzhou Mining Business Group Co., Ltd. between January 2006 and December 2010, as described previously [16]. The cases and controls were selected from the same mines; subjects were excluded if they had clinical evidence of autoimmunity diseases, had received immunosuppressive or immunostimulatory therapy, or were subjected to radiotherapy. The match criteria were as follows: age, dust exposure period, and job type. The questionnaire for participants was conducted by face-to-face interviewers using a double-blind method. This epidemiological questionnaire focus on age, respiratory symptoms, occupational histories, and smoking habits. To confirm diagnoses, high kilovolt chest X-ray and physical examinations were performed based on the China National Diagnostic Criteria for Pneumoconiosis (GBZ 70–2002), which are the same as that of the 1980 International Labour Organization (ILO) in the judgment of opacity profusion [5]. The pneumoconiosis cases were classified into stage I, stage II or stage III according to the size, profusion, and distribution range of opacities. The chest X-rays were assessed by two independent physicians (Z Song and X Jia). Blood samples of 5ml were obtained from all subjects and used for routine laboratory tests. This research protocol was approved by the Institutional Review Board of Nanjing Medical University, and all subjects gave their written informed consent before participating in the study.

### SNPs selection

To select the most likely functional SNPs influencing *SELE* gene, we chose all the non-synonymous SNPs and the SNPs located in 3′UTR and 5′UTR, as determined in the HapMap Genome Browser release (Phase 1 & 2– full dataset). We included the following criteria for SNPs: (i) the SNPs should be located in exon, (ii) the SNPs should be non-synonymous, and (iii) the minor allele frequency (MAF) should be >5% in the Chinese population. T1880C/rs5355 and T1559C/rs5368 were included. We also searched all the SNPs located in 3′UTR and 5′UTR of *SELE* and found that only A16089G/rs4786 has a MAF >5% in Chinese population. Of these three SNPs selected for genotyping, the T1880C/rs5355 and T1559C/rs5368 variants would lead to missense change of the amino acid sequence, and A16089G/rs4786 likely regulates the *SELE* transcription.

### Genotyping

The genomic DNA from peripheral blood lymphocytes was extracted by the conventional phenol-chloroform method, then genotyping data were acquired by the TaqMan method with the ABI 7900HT Real Time PCR system according to the manufacturer's instructions (Applied Biosystem, Foster city, CA, USA). This was accomplished in a blinded fashion without knowledge of the workers' personal details or case status.

The sequences of the primers and probes for each SNP are available on request. Genomic DNA (50 ng) and 0.5× mix was used for each reaction, and amplification was performed under the following conditions: 50°C for 2 min and 95°C for 10 min followed by 45 cycles of 95°C for 15 sec and 60°C for 1 min. Negative controls were included in each plate to ensure accuracy of the genotyping. 10% of the samples were randomly selected for confirmation, and the results were 100% concordant.

### Statistical analyses

Differences in the distributions of demographic characteristics, selected variables, and frequencies of genotypes of *SELE* polymorphisms between the CWP cases and controls were evaluated by use of Student's t-test (for continuous variables) or χ^2^- test (for categorical variables). The Hardy-Weinberg equilibrium (HWE) was tested using a goodness-of-fit χ^2^-test. Unconditional multivariate logistic regression (LR) analyses were accomplished to obtain odd ratios (ORs) and their 95% confidence intervals (95%CIs), with adjustments for possible confounders. For the stratified analysis, the age and dust-exposure cut off used were according to the median ages and dust-exposure years of the recruited patients and controls. Genotypes were coded as wild types (major-allele homozygote) and variants (minor-allele homozygote and heterozygote). The statistical power was calculated by using PS software. (http://biostat.mc.vanderbilt.edu/twiki/bin/view/Main/PowerSampleSize). All statistical tests were two-sided at a significance level of 0.05 and were analyzed with SPSS software (version18.0).

### CART analysis

**Figure 1:**
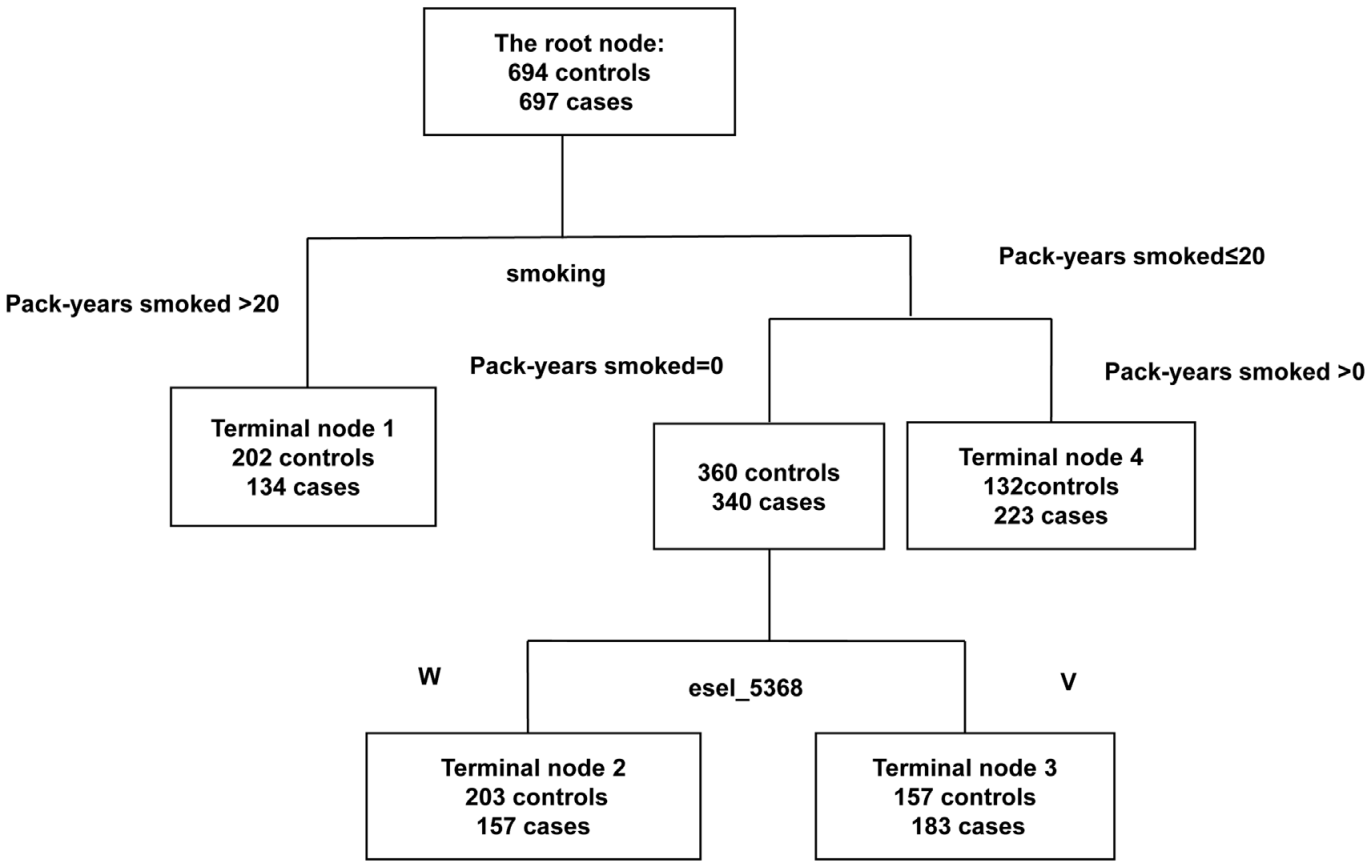
Classification and regression tree analysis of smoking and genetic polymorphism. Terminal nodes show numbers of CWP patients/numbers of controls. Single nucleotide polymorphisms were classified as wild type (W) and variant genotype (V); smoking was defined according to pack-years smoked.

A data mining tool, classification and regression tree (CART), with the rpart package of the R statistical software suite (version 2.11.1) was used. The CART method builds a decision tree via recursive partitioning and automatic selection of optimal cut-off points for variants. Binary recursive partitioning is observed in this classification tree model. A decision tree is a simple and effective method for sifting complex biological data for hidden explicit information. A parent node is split into two child nodes. The models aim to partition, recursively, input variables in order to maximize the purity of a terminal node. A decision tree is created by CART for identifying disease factors and susceptibility alleles and for inferring the mode of inheritance [17]. Figure 1 is a graphic display of decision criteria for each split, which predicts group memberships at the terminal nodes.

### MDR analysis

Another data mining tool, the non-parametric multifactor dimensionality reduction (MDR) software (version 1.1.0) [18], was used to identify the potential locus–locus and gene–environment interactions with trichotomized genotypes and trichotomized pack-years smoked. The fitness of an MDR model was assessed by estimating the testing accuracy and the cross-validation consistency (CVC). Models having a testing accuracy of?0.5 were considered to be true positive. The cross-validations were conducted several times using different random seeds and the results averaged to avoid spurious results due to chance divisions of the data [19]. With a 10-fold cross-validation test, a model was conducted based on 9/10 of the data (training data) and evaluated by the remaining 1/10 of the data (testing data). The CVC was a measure of how many times of 10 divisions of the data that MDR found in the same best model. The sign test counted the number of the cases, k, where the testing accuracy was>0.5 of 10 cross-validation cases. The model with the highest CVC and the highest testing accuracy was selected.

## Results

### Characteristics of the study subjects

The demographic and clinical information is summarized in Table 1. There were no significant differences between the cases and controls in the distribution of age (*P* = 0.103), exposure years (*P* = 0.105), and job types (*P* = 0.534). The distribution of smoking status between cases and controls was parallel (*P* = 0.250), but the smoking amount (pack- years) in CWP cases was significantly less than that of controls (P<0.001). Furthermore, of the cases, the pneumoconiosis stages from I to III were 59.5%, 31.4%, and 9.0%, respectively. The primary information and allele frequencies observed are listed in Table 2. All genotyped distributions of control subjects were consistent with those expected from the Hardy-Weinberg equilibrium. The MAFs of these three polymorphisms were consistent with those reported in the HapMap database.

**Table 1:** Demographic and selected variables among the CWP cases and control subjects.

Variables	CWP (*n* = 697)		Controls (*n* = 694)		*P*
	N	%	N	%	
Age, year (mean ± SD)	68.0±11.1		67.1±8.4		0.103
Exposure years (mean ± SD)	26.6±9.0		27.3±7.8		0.105
Smoking status					
Never	340	48.8	360	51.9	0.250
Ever	357	51.2	334	48.1	
Former	163	23.4	91	13.1	
Current	194	27.8	243	35.0	
Pack-years smoked					<0.001
0	340	49.2	360	52.6	
0–20	223	32.0	132	19.0	
>20	134	19.2	202	29.1	
Work type					0.534
Tunnel and coal mining	663	95.1	652	94.0	
Transport	16	2.3	17	2.5	
Others	18	2.6	25	3.6	
Stage					
I	415	59.5			
II	219	31.4			
III	63	9.0			

**Table 2:** Primary information of genotyped SNPs.

Cluster ID	Region	Function	dbSNP allele	Regulome DB	Protein residue	MAF		HWE^a^
						Case	Control	
rs5355	Exon-13	missense	T/C	7	Phe/Leu	0.043	0.033	0.754
rs 5368	Exon-9	missense	T/C	6	Arg/Ser	0.290	0.273	0.418
rs4786	3'UTR	-	A/G	2b	-	0.413	0.432	0.123

HWE^a^ (Hardy–Weinberg equilibrium) *P* value in the control group.

As shown in Table 3, logistic regression analysis revealed that the *SELE* rs5368 CT genotype, but not the TT genotype, significantly increased the risk of CWP, compared with the CC genotype (OR = 1.28, 95%CI = 1.02–1.60 for CT versus CC; and OR = 0.92, 95%CI = 0.60–1.39 for TT versus CC). However, no significant association with CWP was identified for the other polymorphisms examined. In the stratification analysis (Table 4), significant associations were observed between the *SELE* rs-5368 CT/TT genotypes and patients with stage II CWP (OR = 1.55, 95%CI = 1.12–2.15).

**Table 3:** Distributions of genotypes and their associations with risk of CWP.

Variables	CWP cases		Controls		*P* a	OR(95%CI)	OR(95%CI)^b^
	N	%	N	%			
esel_4786	n = 684		n = 679				
GG	213	31.2	209	30.8		1.0	1.0
AG	377	55.1	353	52.0	0.688	1.05 (0.83–1.33)	1.05 (0.83–1.34)
AA	94	13.8	117	17.2	0.179	0.79 (0.57–1.10)	0.79 (0.57–1.10)
AG/AA	471	68.9	470	69.2	0.886	0.98 (0.78–1.24)	0.99 (0.78–1.24)
G allele	803	58.7	771	56.8		1.0	
A allele	565	41.3	587	43.2	0.309	0.92 (0.79–1.08)	
esel_5368	n = 687		n = 681				
CC	335	48.8	364	53.5		1.0	1.0
CT	305	44.4	262	38.5	0.034	1.27 (1.01–1.58)	1.28 (1.02–1.60)
TT	47	6.8	55	8.1	0.686	0.93 (0.61–1.41)	0.92 (0.60–1.39)
CT/TT	352	51.2	317	46.6	0.083	1.21 (0.98–1.49)	1.22 (0.98–1.51)
C allele	975	71.0	990	72.7		1.0	
T allele	399	29.0	372	27.3	0.316	1.09 (0.92–1.29)	
esel_5355	n = 690		n = 685				
CC	635	92.0	641	93.6		1.0	1.0
CT	50	7.2	43	6.3	0.420	1.17 (0.77–1.79)	1.18 (0.77–1.80)
TT	5	0.7	1	0.1	0.154	5.05 (0.59–43.32)	4.82 (0.56–41.72)
CT/TT	55	7.9	44	6.4	0.267	1.26 (0.84–1.90)	1.27 (0.84–1.91)
C allele	1320	95.7	1325	96.7		1.0	
T allele	60	4.3	45	3.3	0.146	1.34 (0.89–2.03)	

a Two-sided x2 test.

b Adjusted for age, exposure years, pack-years smoked, and job type.

**Table 4:** Stratification analyses between the genotypes of esel_5368 polymorphism and CWP risk.

Variables	Cases/controls	Genotypes (cases/controls)				*P*	OR (95%CI)^a^
		CC		CT/TT			
		n	%	n	%		
Total	687/681	335/364	48.8/53.5	352/317	51.2/46.5	0.083	1.22 (0.98–1.51)
Age							
<68	273/401	132/202	48.4/50.4	141/199	51.6/49.6	0.606	1.08 (0.79–1.48)
≥68	414/280	203/162	49.0/57.9	211/118	51.0/42.1	0.022	1.44 (1.06–1.96)
Exposure years							
<27	266/263	125/118	47.0/44.9	141/145	53.0/55.1	0.624	1.37 (0.97–1.93)
≥27	421/418	210/219	49.9/52.4	211/199	50.1/47.6	0.467	1.11 (0.84–1.45)
Pack-years smoked							
0	338/355	155/200	45.9/56.3	183/155	54.1/43.7	0.006	1.54 (1.14–2.08)
0–20	219/129	112/63	51.1/48.8	107/66	48.9/51.2	0.678	0.90 (0.58–1.40)
>20	130/197	68/101	52.3/51.3	62/96	47.7/48.7	0.854	0.96 (0.62–1.51)
Stage							
I	410/681	212/364	51.7/53.5	198/317	48.3/46.5	0.576	1.05 (0.82–1.34)
II	216/681	95/364	44.0/53.5	121/317	56.0/46.5	0.015	1.55 (1.12–2.15)
III	61/681	28/364	45.9/53.5	33/317	54.1/46.5	0.258	1.56 (0.90–2.70)

a Adjusted for age, exposure years, pack-years smoked, and job type.

To consider potential interactions of cytokine gene polymorphisms on risk of CWP, we combined these three polymorphisms based on the numbers of variant (risk) alleles (i.e., rs4786A, rs5355T, rs5368T). As shown in Table 5, individuals with multiple risk alleles did not have a higher risk of CWP (*P*trend  = 0.731).

**Table 5:** Frequency distributions of the combined genotypes between CWP cases and controls.

No. of risk alleles	Cases (n = 679)		Controls (n = 676)		OR (95%CI)^a^	*P*
	NO	%	No	%		
0–1	322	47.4	307	45.4	1.00	
2	344	50.7	363	53.7	0.91 (0.73–1.13)	0.380
3–4	13	1.9	6	0.9	2.12 (0.79–5.68)	0.148
*P*_trend_						0.731

a Adjusted for age, exposure years, pack-years smoked, and job type.

### Association of multiple factor interaction with CWP risk (CART analysis)

CART analysis was used to predict the interactions among risk factors of CWP. Interactions between three environment factors (exposed years, pack-years smoked, and job type) and the three SNPs were explored. The final tree structure contained four terminal nodes within rs5368 and pack-years smoked. Figure 1 showed that the initial split of the root node was pack-years smoked, indicating that, among the factors considered, smoking was the strongest risk factor for CWP. In non-smoking individuals, however, *SELE* rs_5368 was the most significant risk factor, and carriers of the *SELE* rs_5368 variant genotype had a 1.54-fold increased CWP risk with a 54% case rate (OR = 1.54; 95% CI = 1.11–2.05).

### Association of multiple factor interaction with CWP risk (MDR analysis)

The MDR method was used to assess potential locus–locus and gene–environment interactions with three SNPs and three environment factors. As shown in Table 6, pack-years smoked was the strongest factor for predicting CWP risk with the highest CVC (100%) and testing accuracy (56.49%). We also observed that the two-factor model including smoking and *SELE* rs_5368 was the most accurate model (57.52%), with a perfect CVC of 10 that was statistically significant (*P* = 0.0107). Models including three or four factors showed decreases in testing accuracy and CVC.

**Table 6:** MDR analysis for the CWP risk predication.

Best Model	Training. Bal. Acc	Testing. Bal. Acc	Sign test (P)	CVC
One factor				
Pack-years smoked	0.5649	0.5649	9 (0.0107)	10/10
Two factor				
Pack-years smoked	0.5885	0.5752	9 (0.0107)	10/10
esel _5368				
Three factor				
Pack-years smoked	0.6008	0.5674	9 (0.0107)	7/10
esel_5368				
tage				
Four Factor				
Pack-years smoked	0.6204	0.5622	9 (0.0107)	10/10
esel_5368				
esel_4786				
tage				

## Discussion

Genetic and environmental factors are involved in the development of CWP. In this case-control study, we explored, in a Chinese population, associations between three functional polymorphisms in *SELE* and the risk of CWP and the interaction between genetic and environmental factors. Of three SNPs (*SELE* rs_4786, *SELE* rs_5355, and *SELE* rs_5368), the *SELE* rs5368 CT genotype was associated with increased risk of CWP (OR = 1.28, 95% CI = 1.02–1.60), and the interaction between rs_5368 and smoking was detected. The fact that an association was found with a heterozygote genotype but not with the homozygote may be due to the small sample size. This also indicates that there might be a threshold effect for the production of the E-selectin protein or problems associated with heterodimer activity.

Previous studies on the association between *SELE* rs_5368 and soluble E-selectin (sE-selectin) levels have been controversial. Chen et al. [20] revealed significant association of *SELE* rs_5368 with soluble sE-selectin level in a Chinese population while Miller et al. [21] found no evidence of association between the *SELE* rs_5368 and circulating sE-selectin level. Interestingly, a recent study conducted in Taiwanese individuals aimed to elucidate the association between *SELE* SNPs and the plasma levels of sE-selectin and metalloproteinases 9 (MMP9). This study provided a compelling story that the minor alleles of rs5368 were significantly associated with higher plasma level of MMP9 [22]. In CWP, collagen degradation does not keep pace with collagen production, resulting in extracellular accumulation of fibrillar collagen. MMPs are responsible for extracellular collagen degradation by recognition of specific cleavage sites that have characteristic imino acid content [23], [24]. In addition, MMP-9 can further degraded fragments of cleaved fibrillar collagen [25]. Thus, the *SELE* rs_5368 may exert influence over the plasma levels of sE-selectin and MMP9 to participate in the pathogenesis of CWP.

Besides referring to the existing researches, we also exploited Exonic splicing enhancers (ESEs) finder, (release2.0: http://exon.cshl.edu/ESE/) which allows scanning of nucleotide sequences to predict putative ESEs responsive to the human Ser/Arg-rich proteins (SR proteins) SF2/ASF, SC35, SRp40 or SRp55 [26]. The searching result of rs_5368 in ESE finder namely shows that the rs5368 C allele forms two motifs which could bind separately to SRp40 and SRp55, while the rs5368 T allele binds only to SRp55 [27]. Thus, the *SELE* rs_5368 may influence the splicing of *SELE* and generate different splice variants of *SELE*.

Cell adhesion molecules are involved in the development of various inflammatory diseases [28]. In general, inhibition or loss of cell adhesion molecules attenuates the inflammatory response in experimental models [29]–[32]. The selectin family consists of three cell-surface molecules expressed by leukocytes (L-selectin), vascular endothelium (E-selectin), and platelets (P-selectin). E-selectin expression is induced within several hours after activation with inflammatory cytokines. Inhibition or loss of E-selectin leads to a reduction in neutrophil rolling and to acute emigration in models of inflammatory, such as the Arthus reaction, dermal inflammation, and peritonitis models [33]–[35].

Yoshizaki et al [7] showed that, in bleomycin-induced mice, fibrosis was evident in mice lacking adhesion molecules. L-selectin deficiency inhibited lung fibrosis, but P-selectin deficiency and E-selectin deficiency augmented the fibrosis [29]. Another study involving E-selectin −/−, P-selectin −/− mice also revealed an inhibitory role of E-selectins in the development of bleomycin-induced pulmonary fibrosis [36]. Both studies conducted flow cytometric analysis between cell adhesion molecule-deficient mice treated with bleomycin and WT mice and found that loss of cell adhesion molecule function selectively altered the trafficking pattern of fibrogenic Th2 and Th17 cells and anti-fibrogenic Th1 cells to the lung. E-selectin loss may induce dominant Th2 and Th17 cell infiltration. This pattern of leukocytes in the BAL resulted in differential production of cytokines. Lack of E-selectin reduced IFN-γ and increased IL-4, IL-6, IL-17, and TGF-β1 [29], [37], [38]. IFN-γ, one of the Th1 cytokines, has an antifibrotic effect on pulmonary fibrosis [39]. In addition, we should pay attention to natural killer T (NKT) cells which are innate memory cells. NKT cells plays important roles in the initiation and regulation of the immune response. Horikawa M et al [36] revealed that NKT cell infiltration into the lung was dependent on E-selectin expression. These results provide additional clues to understanding the complexity of the pathogenesis of pulmonary fibrosis.

To evaluate the contribution of genetic and environmental factors in CWP risk in the present study, data mining approaches were used. The first split in CART and the best one-factor model in MDR both indicated that smoking was the predominant risk factor for CWP. In the CART analysis, we found that the *SELE* rs_5368 polymorphism significantly interacted with pack-years smoked; a similar result was obtained by the MDR method. For non-smokers, the CWP risk was attributable more to the *SELE* rs_5368 variant genotype. Use of CART and MDR analysis to explore the interaction function is a major strength in our study. For fibrosis, a common but complex multifactorial diseases, interpreting interactions between genetic factors and the environment is a challenge. Traditional parametric statistical methods, such as LR analysis, are of limited use and could result in an increase of type I errors and a decreased power in detecting interactions [18]. Furthermore, we used the CART and MDR methods, which can reduce the chances of making type I errors and improve the statistical power, to identify potential gene–environment interactions [40], [41].

Several limitations of this study should be addressed. First, since this was a population-based, case-control study, we could not rule out the possibility of selection bias of subjects. Second, our sample size was only moderate, further studies are required for replicating our results in larger and different ethnic populations. Third, we selected all the eligible SNPs in UTR region and non-synonymous SNPs rather than a tagging SNP approach. Thus, we did not evaluate the entirety of polymorphic variations across *SELE*. Additional representative SNPs should be included to identify useful markers to predict the risk of CWP. Finally, Since three SNPs were tested, we should apply an appropriate multiple testing correction, such as the Bonferroni correction, otherwise the significant association between the SELE rs_5368 polymorphism and CWP risk should be interpreted with caution.

In conclusion, the present study indicates that the functional *SELE* rs_5368 polymorphism, which interacts with smoking, is associated with an increased risk of CWP in a Chinese population. Further functional researches and validation studies with diverse populations are warranted to confirm our findings.
